# Comparative Metagenomics Reveals Microbial Signatures of Sugarcane Phyllosphere in Organic Management

**DOI:** 10.3389/fmicb.2021.623799

**Published:** 2021-03-22

**Authors:** Ahmad Nuruddin Khoiri, Supapon Cheevadhanarak, Jiraporn Jirakkakul, Sudarat Dulsawat, Peerada Prommeenate, Anuwat Tachaleat, Kanthida Kusonmano, Songsak Wattanachaisaereekul, Sawannee Sutheeworapong

**Affiliations:** ^1^School of Bioresources and Technology, King Mongkut’s University of Technology Thonburi, Bangkok, Thailand; ^2^Pilot Plant Development and Training Institute, King Mongkut’s University of Technology Thonburi, Bangkok, Thailand; ^3^Biochemical Engineering and Systems Biology Research Group, National Center for Genetic Engineering and Biotechnology, National Science and Technology Development Agency at King Mongkut’s University of Technology Thonburi, Bangkok, Thailand; ^4^Bioinformatics and Systems Biology Program, School of Bioresources and Technology, King Mongkut’s University of Technology Thonburi, Bangkok, Thailand; ^5^Faculty of Food Industry, King Mongkut’s Institute of Technology Ladkrabang, Bangkok, Thailand

**Keywords:** agricultural shift, microbial signatures, phyllosphere, sugarcane, farming practices

## Abstract

Converting conventional farms to organic systems to improve ecosystem health is an emerging trend in recent decades, yet little is explored to what extent and how this process drives the taxonomic diversity and functional capacity of above-ground microbes. This study was, therefore, conducted to investigate the effects of agricultural management, i.e., organic, transition, and conventional, on the structure and function of sugarcane phyllosphere microbial community using the shotgun metagenomics approach. Comparative metagenome analysis exhibited that farming practices strongly influenced taxonomic and functional diversities, as well as co-occurrence interactions of phyllosphere microbes. A complex microbial network with the highest connectivity was observed in organic farming, indicating strong resilient capabilities of its microbial community to cope with the dynamic environmental stressors. Organic farming also harbored genus *Streptomyces* as the potential keystone species and plant growth-promoting bacteria as microbial signatures, including *Mesorhizobium loti*, *Bradyrhizobium* sp. SG09, *Lactobacillus plantarum*, and *Bacillus cellulosilyticus*. Interestingly, numerous toxic compound-degrading species were specifically enriched in transition farming, which might suggest their essential roles in the transformation of conventional to organic farming. Moreover, conventional practice diminished the abundance of genes related to cell motility and energy metabolism of phyllosphere microbes, which could negatively contribute to lower microbial diversity in this habitat. Altogether, our results demonstrated the response of sugarcane-associated phyllosphere microbiota to specific agricultural managements that played vital roles in sustainable sugarcane production.

## Introduction

Over the past decades, conventional farming practice that mainly relies on agrochemical inputs has significantly increased the global *per capita* agricultural production (Pretty, 2008). However, this effort not only raises production costs but also causes serious problems to the environment including soil contamination and degradation, emission of fertilizers and pesticides, loss of biodiversity, and several negative impacts on human health (Rivera et al., 2017). Therefore, alternative methods are needed for improving agricultural outputs or reducing dependency on agrochemicals and equilibrating productivity with sustainability.

Converting conventional farms to organic management systems, known as transition farming, could potentially be a solution to improve biodiversity and sustainably increase food production (Gonthier et al., 2014). However, how long it takes to recover the loss of biodiversity and functionality caused by unsustainable farming practices remains questionable. Organic farming is one kind of farming practice intended to reduce the need for chemical inputs to improve overall ecosystem health. It relies on animal waste, green manure, biological pest control, and some techniques including crop rotation and multiple cropping to enhance soil nutrients and crop fertility (Pimentel et al., 2005). Compared to conventional farming, organic farming is much closer to natural ecosystems because it depends less on agrochemicals and increases more on nutrient recycling systems. Hence, this farming has been shown to be more effective in reducing the negative impacts of agricultural practices on the environment and promoting biodiversity (Bengtsson et al., 2005). Previous studies have demonstrated that organic farming could promote more diverse below-ground microbial communities (i.e., soil and root niches) compared with conventional farming (Lupatini et al., 2017; Hartman et al., 2018). Another research also showed that organic fertilization improved the abundance of microbial nitrogen (N)-cycle genes, which were associated with N availability in the soil (Morugán-Coronado et al., 2019).

Phyllosphere, the above parts of plant compartments dominated by leaves, is densely colonized by numerous microbial taxa such as bacteria, fungi, and yeasts (Müller and Ruppel, 2014). Phyllosphere microbial community has been known to participate in nutrient cycles, e.g., carbon (Knief et al., 2012) and nitrogen (Fürnkranz et al., 2008), and degradation of air pollutants (Ali et al., 2012). Microbes residing in this environment have also been thought to play important roles in plant health and ecosystem productivity (Laforest-Lapointe et al., 2017). For instance, microbial colonizers can promote the growth of their host through phytohormone production (Taghavi et al., 2009), foliar pathogen protection (Ritpitakphong et al., 2016), and stress tolerance enhancement (Redman et al., 2002). Besides, as an open system, the phyllosphere habitat can be easily invaded by exogenous microbes through transmission from the atmosphere, soil, and insects (Vorholt, 2012; Maignien et al., 2014), including phytopathogens. Thus, maintaining homeostasis of phyllosphere microbiota will be critical for the survival of the host plant (Chen et al., 2020). With all these traits, harnessing beneficial phyllosphere microbiota as new alternatives of biofertilizers, biopesticides, or biostimulants seems to be a promising target for improving plant and environmental fitness, which leads to sustainable agriculture to feed the continuously growing global population. However, a comprehensive understanding of phyllosphere microbial diversity and functionality in response to agricultural shift remains to be elucidated and will be an important step to achieve these goals.

Sugarcane (*Saccharum officinarum* L.) is a C4 grass plant that has approximately 50% higher photosynthesis efficiency than C3 plants (Kajala et al., 2011). It is also one of the most economically important crops used as a major source for sugar and bioethanol production (Waclawovsky et al., 2010). This plant is grown in tropical and subtropical countries in which Brazil is the world’s largest producer, followed by India, China, and Thailand (FAO, 2018). However, to increase sugarcane yield, a high rate of synthetic N fertilizers is commonly applied as well as pesticides for plant protection against phytopathogen infections (Robinson et al., 2011), resulting in ecological damages. Besides, previous sugarcane microbiome studies have mainly focused on the below-ground compartments (i.e., soil, rhizosphere, and root) using amplicon sequencing such as 16S rRNA and ITS barcoding regions (Paungfoo-Lonhienne et al., 2014, 2015; Dong et al., 2018), which are limited to the taxonomic classification at the genus level and are biased by the PCR amplification process. Moreover, the important roles of phyllosphere microbial community have been less explored than those of other niches, especially in correlation with farming management practices, despite their potential benefits on agricultural productivity.

Previous studies have demonstrated that organic farming promoted higher species richness of wheat phyllosphere fungal microbiome than conventional farming (Karlsson et al., 2017). Moreover, different agricultural inputs in organic and conventional farms would directly and/or indirectly influence the phyllosphere microbial community. Karlsson et al. (2014) showed that fungicides negatively affected fungal community composition on wheat leaves. Perazzolli et al. (2014) reported that the richness and diversity of bacterial and fungal communities of graphene leaves were altered by penconazole fungicide and biological control agent (*Lysobacter capsici* AZ78) treatments. Similarly, Cernava et al. (2019) exhibited that pest and pathogen management practices, i.e., pesticides and biological treatments, shaped the microbial population of tea leaves. In addition, Xiang et al. (2020) suggested that functional traits of phyllosphere microorganisms were more sensitive to land use and anthropogenic disturbance compared with soil microbiota. Thus, we hypothesized that different farming practices would have impacts on the taxonomic diversity and functional capacity of the above-ground microbial community and promote distinct microbial profiles with unique signatures.

To gain deeper insights into how taxonomic diversity and functional capacity of phyllosphere microbial community are shaped by farming practices, we performed shotgun metagenome sequencing on sugarcane leaves collected from organic farming where a bio-compost and swine manure were applied as input and conventional farming that has long been treated with agrochemicals. We also included transition farming that had been switched to the organic system 2 years before our sample collection to observe how phyllosphere microbes are involved in this transformation process. We aimed to observe structural changes of phyllosphere microbiota regarding agricultural management types and identify beneficial microbes that could be potentially used as biostimulants to replace agrochemicals to transform the conventional practices toward organic systems for more sustainable farming.

## Materials and Methods

### Study Area, Sample Collection, and Storage

In this study, sugarcane leaves (plant age: 10 months old; maturing; and ripening stage) were sampled in April 2019 from three different farming practices in Thailand. These sampling sites included organic farming (OP) at Rai Sukphoang, Chom Bueng District, Ratchaburi (13°35′42.5″N 99°34′58.0″E); transition farming (TP) at Rai Sarot, Rang Bua, Chom Bueng District, Ratchaburi (13°37′52.8″N 99°33′10.7″E); and conventional farming (CP) at Rai Pramote, Photharam District, Ratchaburi (13°44′34.2″N 99°54′16.6′E) (Supplementary Table 1 and Supplementary Figure 1). In total, nine samples of sugarcane Khon Kaen 3 (KK3) cultivar were collected for three replicates (i.e., three different sugarcane tillers from the same field) per farm. Based on Kuijper’s leaf numbering system (Kuijper, 1915), leaves number +1 to +4 were chosen, and the leaf sheath and the leaf tip were removed using a surface-sterilized scissor. All samples were then stored in a sterile zipper locking plastic bag and kept cool during field collection and transported at 4∘C, then frozen at −80∘C until used for downstream processes.

### DNA Extraction

Sugarcane leaf samples were ground using mortars and pestles under liquid nitrogen to accelerate the grinding process. Phyllosphere microbial DNA from 0.2 *g* of leaf powder was extracted using the innuPREP Plant DNA Kit (AnalytikJena AG, Berlin, Germany) according to the manufacturer’s instructions. The DNA was eluted in a Tris-EDTA (TE) buffer, then stored at −80∘C for subsequent steps. Total genomic DNA samples were qualitatively and quantitatively measured by using gel electrophoresis and a NanoDrop spectrophotometer (Thermo Scientific, Wilmington, DE, United States), respectively. The obtained phyllosphere microbial DNA samples comprise both epiphytic and endophytic microbial DNA.

### Library Preparation and Sequencing

Phyllosphere microbial DNA samples were sent for sequencing to ZymoBIOMICS^®^ Shotgun Metagenomic Sequencing Service (Zymo Research Corp., Irvine, CA, United States) assisted by S. M. Chemical Supplies Co., Ltd., Thailand. Briefly, a metagenome sequencing library was prepared from up to 100 ng of genomic DNA using Nextera^®^ DNA Flex Library Prep Kit (Illumina, San Diego, CA, United States) according to the manufacturer’s instructions. The library was tagged with internal dual-index 8-bases barcodes and Nextera^<®^ adapters (Illumina, San Diego, CA, United States), then quantified using TapeStation^®^ (Agilent Technologies, Santa Clara, CA, United States) and equally pooled. The resulting DNA pool was measured using quantitative PCR (qPCR). In addition, a negative control (i.e., blank library preparation control) was used during the metagenome library preparation process. Finally, 11 metagenome libraries including a mock sample (ZymoBIOMICS^TM^ Microbial Community DNA Standard II, Catalog No. D6311) were sequenced on Illumina HiSeq 1500 platform paired-end sequencing (2 × 100 bp).

### Metagenomic Analysis

#### Quality Control and Host DNA Removal

In order to maximize the quality of the datasets, the following steps were performed. First, sequence reads were subjected to quality checking using FastQC version 0.11.9 (Andrews, 2010) and MultiQC version 1.8 (Ewels et al., 2016). Furthermore, the remaining adapters and the low quality of reads with Phred score less than 15 were trimmed, and the short reads less than 50 bp were discarded using Trimmomatic version 0.39 (Bolger et al., 2014) with the parameters as follows: NexteraPE-PE.fa:2:30:10:2:keepBothReads, SLIDINGWINDOW 4:15, and MINLEN: 50. High-quality reads were subsequently aligned with the publicly available sugarcane genome (*Saccharum spontaneum* cultivar AP85-441: GenBank accession number: GCA_003544965.1, downloaded as of March 24, 2020) using Bowtie2 version 2.3.5.1 (Langmead and Salzberg, 2012) to identify and filter out host contaminant sequences.

#### Taxonomic Profiling and Diversity Analysis

Non-host sequences were taxonomically assigned using Kraken2 version 2.0.8-beta (Wood et al., 2019) against a custom Kraken2 database consisting of archaeal, bacterial, fungal, plasmid, and viral sequences (downloaded from NCBI RefSeq as of August 15, 2020). Bracken version 2.5.0 (Lu et al., 2017) was further used to re-estimate the microbial abundance at the species level. The microbial abundance table generated from metagenome taxonomic profiling with Kraken2 + Bracken was used as an input for diversity analyses. These were performed on R program version 3.6.2 (R Core Team, 2019) with *vegan* version 2.5-6 (Oksanen et al., 2019), *ggplot2* version 3.3.0 (Wickham, 2016), and *phyloseq* version 1.28.0 (McMurdie and Holmes, 2013) packages. Briefly, a one-way ANOVA test was computed based on a Shannon estimator to determine the species diversity and the Pielou index for quantifying species evenness in each sample (α-diversity). Furthermore, the differences of overall microbial profiles among farming practices (β-diversity) were estimated by performing unconstrained principal coordinate analysis (PCoA) using the Bray–Curtis distance. The statistical significance of β-diversity was then determined by PERMANOVA test with 999 permutations, followed by a multivariate homogeneity of group dispersions (variances) test (BETADISPER) to check whether the investigated groups are homogeneously dispersed in relation to their microbial taxa. Geographical location (district) was also included in these statistical assessments of β-diversity as a potential confounding effect. In addition, the adequacy of sampling was quantified by calculating Good’s coverage index and by plotting an α-rarefaction curve.

#### Metagenome Functional Annotation

Functional analysis of metagenome was performed with sequence similarity searches using SqueezeMeta version 1.2.0 with function “sqm_reads.pl” (Tamames and Puente-Sánchez, 2019). In brief, sequence reads were functionally assigned against the Kyoto Encyclopedia of Genes and Genomes (KEGG) Orthology (KO) database, downloaded as of August 18, 2020 (Kanehisa and Goto, 2000; Kanehisa et al., 2016) using the DIAMOND (blastx) algorithm (Buchfink et al., 2015) with default options (maximum *E*-value = 1e-03 and minimum percent identity = 50). Finally, the function of fun3 in the SqueezeMeta program was employed for functional assignments. In addition, only sequences that were previously assigned as archaea, bacteria, fungi, and viruses were kept for functional analyses. Thus, the other sequences that could not be assigned for both taxonomy and function were discarded.

The generated KO abundance table was subsequently preprocessed following the workflow of the previous study (Yurgel et al., 2019). First, the count matrix was normalized to the Reads Per Kilobase per Genome equivalent (RPKG) method using MicrobeCensus version 1.1.0 (Nayfach and Pollard, 2015). MinPath (Ye and Doak, 2009) and the function “pathway_pipeline.py” in PICRUSt2 version 2.3.0-b (Douglas et al., 2020) were then employed to map a normalized KO table to KEGG pathways with the “–no_regroup” setting. The predicted pathways were further manually grouped into the levels of KEGG BRITE hierarchy termed as the KEGG category and the KEGG pathway. Furthermore, gene diversity at the KO level was estimated using the Shannon index. Functional β-diversity was calculated by projecting the data onto the unconstrained PCoA plot using Bray–Curtis dissimilarity, followed by PERMANOVA with 999 permutations and BETADISPER tests, in which farming practices and districts were the main and confounding effects, respectively. In addition, one-way ANOVA was used to statistically test whether metabolic processes at the level of the KEGG category and the KEGG pathway were affected by farming practices.

#### Core Feature Identification and Differential Abundance Analysis

Core taxonomic and functional metagenomes were quantified using the “core_members” function in R package *microbiome* version 1.6.0 (Lahti and Shetty, 2019) with the following criteria: detection = 0.001 and prevalence = 95%. Furthermore, the differential abundance of taxonomic and functional metagenomes among farming practices was statistically examined using the linear discriminant analysis (LDA) effect size (LEfSe) method as implemented in the LEfSe version 1.0.8 (Segata et al., 2011). LEfSe employs a non-parametric Kruskal–Wallis sum-rank test to detect any differentially abundant feature between groups. Biological consistency is then examined with a set of pairwise tests using an unpaired Wilcoxon rank-sum test. Finally, the effect size of significantly abundant features is estimated using the LDA test. In this study, LEfSe parameters such as the LDA score and the Wilcoxon *p*-value were set to 2.0 and 0.05, respectively. The results were then visualized in bar graphs using R package *ggplot2* version 3.3.0 (Wickham, 2016).

#### Microbial Co-occurrence Network Construction

A microbial co-occurrence network was constructed based on the Sparse Correlations for Compositional data (SparCC) algorithm (Friedman and Alm, 2012) using FastSpar version 0.0.10 (Watts et al., 2018). The microbial abundance matrix, in which rows and columns represent taxa and samples, respectively, was used as an input and was preprocessed as follows. First, the data were divided into three subsets based on their origin (Organic, Transition, and Conventional). Microbial species (features) present in less than 2 out of 3 samples and the abundances lower than 11 were removed out from the count table. To avoid comparison biases caused by unequal numbers of features after the filtering step, only 500 features of each filtered dataset were randomly selected for network analysis. Empirical SparCC correlation coefficients (*p*-value) were calculated by a bootstrapping method with 1,000 iterations, and three farming-specific co-occurrence networks were then constructed. Cytoscape version 3.8.0 (Shannon et al., 2003) was employed to visualize the networks with the following criteria: strong correlation (| *r*| > 0.6) and *p*-value < 0.05. In addition, the Girvan–Newman algorithm was executed to find network modules using the Cytoscape app *clusterMaker2* version 1.3.1 (Morris et al., 2011), while other network topological features were examined using available functions in R package *igraph* version 1.2.4.2 (Csardi and Nepusz, 2006). Microbial taxa (nodes) with degree > 10 and betweenness centrality < 0.1 were considered as the hub species as explained in the previous research (Berry and Widder, 2014). Node attributes of each network were quantified by a bootstrapping method with 10,000 iterations and were subsequently compared with the two-sample Kolmogorov–Smirnov test using the “ks.test” function in the *stats* R package.

## Results

### General Information of Metagenome Dataset

A total of 118,535,073 raw sequence reads from 9 shotgun metagenome libraries were generated by the Illumina platform, ranging from 9,892,628 to 29,216,274 reads per sample. The mean sequence length of paired-end reads was 100 bp with an average Phred score above 30. After quality-filtering, i.e., host decontamination and base quality trimming, approximately 93% of reads were discarded, remaining 8,176,818 high-quality sequences for subsequent analyses (Supplementary Table 2). In addition, around 250,000 reads could be taxonomically assigned belonging to archaeal, bacterial, fungal, and viral species.

### Taxonomic Composition of Sugarcane Phyllosphere Microbiota

**FIGURE 1:**
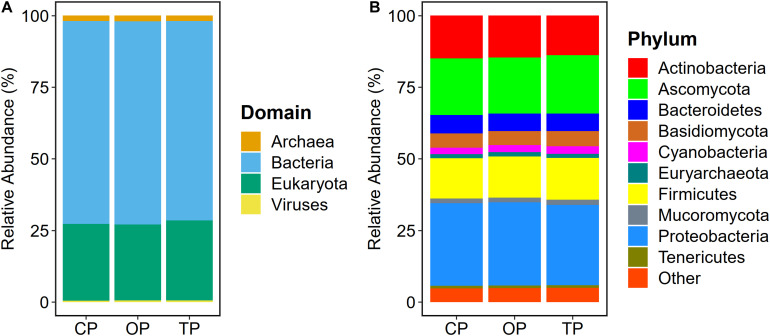
Microbial taxonomic composition of sugarcane phyllosphere. Relative abundance at **(A)** domain and **(B)** phylum levels in conventional (CP), organic (OP), and transition (TP) farming practices.

Read-based metagenome taxonomic profiling on Kraken2 followed by microbial abundance re-estimation with Bracken yielded 6,572 overall microbial taxa across all samples. Particularly, 5,488 bacterial species (OP: 5,255; TP: 4,968; and CP: 4,945), 331 fungal species (OP: 330; TP: 330; and CP: 331), 287 archaeal species (OP: 272; TP: 252; and CP: 251), and 466 viral species (OP: 319; TP: 218; and CP: 189) were observed. At the domain level, bacteria (70.6%) were the most abundant microbial colonizers inhabiting sugarcane phyllosphere habitat, followed by eukaryotic, i.e., fungal (27%), archaeal (1.8%), and viral (0.6%) communities (Figure 1A). On the level of phylum, Proteobacteria, Ascomycota, Actinobacteria, Firmicutes, Bacteroidetes, Basidiomycota, Cyanobacteria, Mucoromycota, Euryarchaeota, and Tenericutes were the top 10 predominant microbial phyla (Figure 1B).

### Functional Potentials of Sugarcane Phyllosphere Microbiota

In total, 3,797 KEGG orthologs (KOs) consisting of 185 KEGG pathways and 36 KEGG categories were observed across all samples. Gene functions related to metabolism were the most abundant, accounting for 69.4% of overall annotated sequences (Supplementary Figure 2). Specifically, amino acid metabolism (12.1%), energy metabolism (11%), carbohydrate metabolism (9.4%), glycan biosynthesis and metabolism (8.1%), biosynthesis of other secondary metabolites (7.3%), metabolism of terpenoids and polyketides (5.3%), lipid metabolism (4.8%), and metabolism of cofactors and vitamins (4.8%) were among the top KEGG functional categories (Supplementary Figure 3). At the level of the KEGG pathway, other glycan degradation (ko00511) was the predominant pathway, followed by photosynthesis (ko00195), valine, leucine, and isoleucine biosynthesis (ko00290), phenylpropanoid biosynthesis (ko00940), aminoacyl-tRNA biosynthesis (ko00970), oxidative phosphorylation (ko00190), and biosynthesis of ansamycins (ko01051), complementing the dominant pathways with abundances higher than 2% (Supplementary Figure 4).

### Farming Practices Significantly Induced Distinct Taxonomic and Functional β-Diversities

**FIGURE 2:**
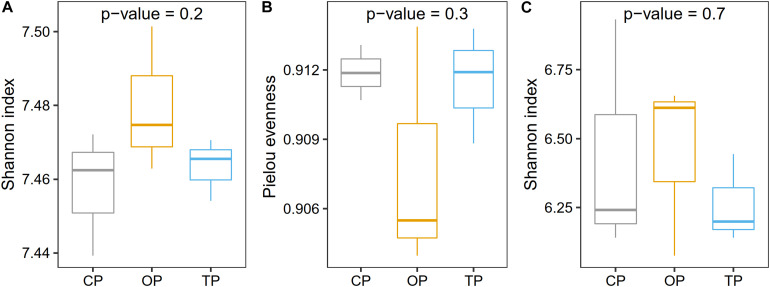
Effect of farming practices on taxonomic α-diversity determined using **(A)** the Shannon index and **(B)** Pielou evenness, and **(C)** microbial gene diversity estimated with the Shannon index. Statistical analysis was performed on one-way ANOVA.

Prior to taxonomic diversity analyses, the number of reads was rarefied to 19,000 for each sample to avoid comparison biases caused by an unequal library size (Supplementary Figure 5). After this process, an average Good’s coverage was reported as 95% (Supplementary Table 3), indicating that the sample size was still enough for downstream analyses. Species diversity was the highest in organic farming and gradually decreased in transition and conventional farms, respectively (Figure 2A), while the evenness index showed the opposite trend with organic farming being the lowest (Figure 2B). Similarly, functional diversity on the gene level using the Shannon index also revealed that organic farming had the greatest microbial functions than the other two farming practices (Figure 2C). However, no statistical significance was observed for α-diversity estimation.

**FIGURE 3:**
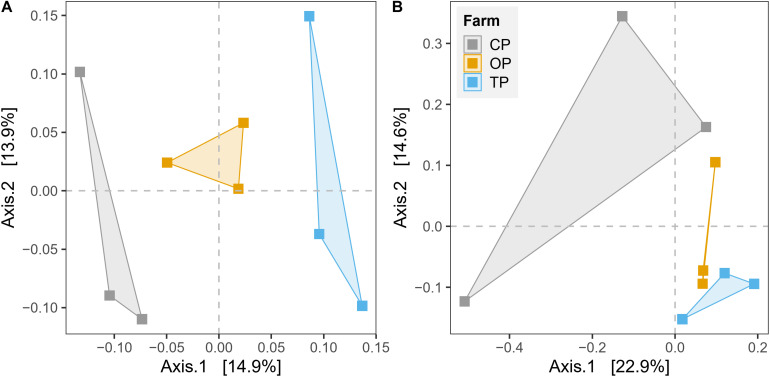
Unconstrained principal coordinate analysis (PCoA) based on Bray–Curtis distance for the effect of agricultural practices on **(A)** taxonomic diversity and **(B)** gene function.

β-Diversity was estimated by plotting unconstrained PCoA and executing PERMANOVA test based on Bray–Curtis dissimilarity on the level of species and KO for taxonomy and functional potential, respectively. The PCoA plots showed that microbial communities and their gene functions were clearly separated along the first principle coordinate according to types of farming practices (Figure 3). PERMANOVA results further confirmed the observed differences among the three microbial habitats (*R*^2^ = 0.27, *p* < 0.01 and *R^2^* = 0.28, *p* < 0.05 for taxonomic and functional diversity, respectively) (Supplementary Table 4). However, the significant output of the BETADISPER test in taxonomic profile (*p* < 0.01), but not in functional potential (*p* > 0.05), suggested that differences of taxonomic β-diversity might be also slightly influenced by within-group dispersion (Supplementary Table 4). The PERMANOVA analysis also provided the top 50 most influential taxa, which potentially contribute to the differentiation of taxonomic profile regarding farming practices (Supplementary Figure 6). In addition, we also found a significant effect of the district (geographical location) from the PERMANOVA test of both taxonomic β-diversity (*R*^2^ = 0.14, *p* < 0.05) and functional β-diversity (*R^2^* = 0.17, *p* < 0.05) (Supplementary Table 4).

### Cell Motility and Energy Metabolism Significantly Depleted Under Conventional Farming

**FIGURE 4:**
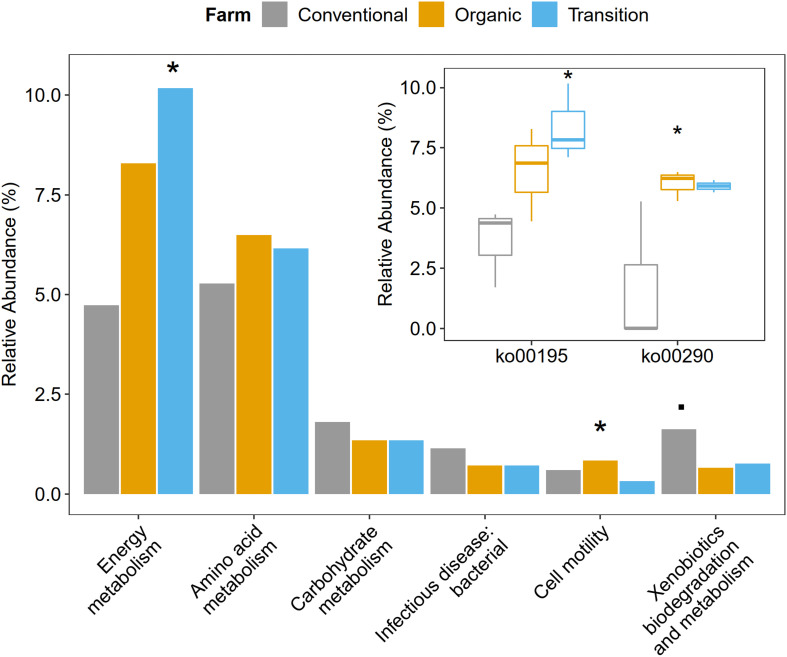
The impacts of farming practices on metabolic processes at the second and third levels of KEGG hierarchy. The statistical test was performed using one-way ANOVA in which *p*-values < 0.05 were considered significant as represented by the (*) symbol, while the (.) character reflected *p*-value < 0.1.

Functional metagenome analysis at the level of the KEGG category and the KEGG pathway showed that microbial gene abundances related to cell motility and energy metabolism were significantly affected by farming practices. Particularly, the abundance of cell motility was the highest in organic farming (ANOVA, *p* < 0.05). In terms of energy metabolism, transition farming was prominent (ANOVA, *p* < 0.05), specifically the photosynthesis pathway (ko00195) (ANOVA, *p* < 0.05). For amino acid metabolism, both transition and organic farms were slightly higher than conventional farming (ANOVA, *p* > 0.1). Specifically, the valine, leucine, and isoleucine biosynthetic pathway (ko00290) was significantly predominant in organic farming and was the lowest in the conventional one (ANOVA, *p* < 0.05). Further, conventional farming hosted lightly greater numbers of microbial genes associated with carbohydrate metabolism (ANOVA, *p* > 0.1), xenobiotics biodegradation and metabolism (ANOVA, *p* < 0.1), and infectious disease: bacterial (ANOVA, *p* > 0.1) (Figure 4 and Supplementary Figures 3, 4).

### Organic Farming Harbored a Complex Phyllospere Microbial Network With Streptomyces as the Major Hub Taxa

**FIGURE 5:**
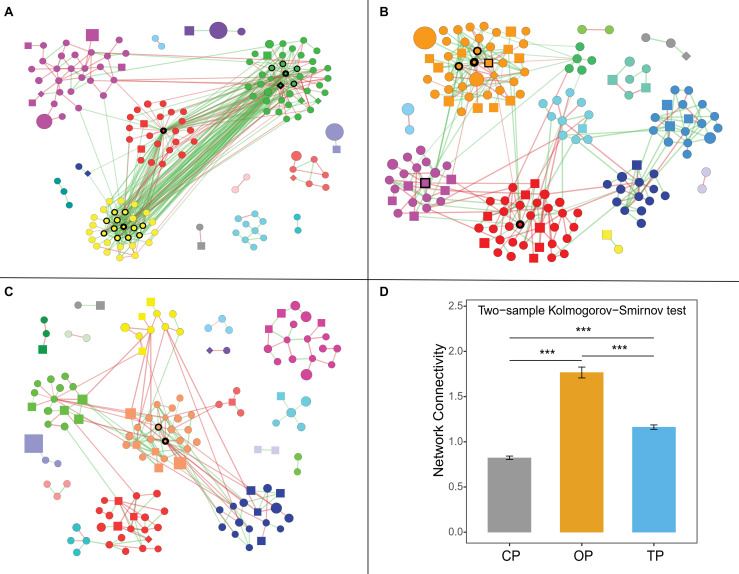
Farming-specific co-occurrence networks of **(A)** organic, **(B)** transition, and **(C)** conventional farming practices. Green and red colors of the edges represent positive and negative relationships, respectively, while the color of nodes demonstrates network modules. The shape of vertices reflects microbial domains (i.e., circle: bacteria; square: fungi; and diamond: archaea), and the size of nodes refers to abundances. In addition, the nodes with black borders were assigned as hub taxa, and the width of the border reflects the node degree score. Note: only nodes with at least 1 connection are visualized. **(D)** Network connectivity or node degree represents the number of edges connected to a node. The symbol (***) represents statistical significance of *p*-value < 0.0001.

Co-occurrence network analysis showed that organic farming harbored the greatest network connectivity (Figure 5), number of edges, and network density (Supplementary Table 5). Similarly, the number of hub species identified was the highest in organic farming (19 species) compared to 6 and 2 species in transition and conventional farms, respectively (Supplementary Figure 7). The hub species of the organic network was dominated by 4 bacterial species belonging to the genus *Streptomyces*, followed by nitrogen-fixing bacteria [i.e., carotenoid-producing *Calothrix* sp. 336/3 (Kosourov et al., 2016), *Paraburkholderia* sp. CCGE1002 (Ormeño-Orrillo et al., 2012), and *Azoarcus* sp. DD4 (Deng et al., 2019)], polysaccharide-degrading *Lacinutrix* sp. 5H-3-7-4 (Klippel et al., 2011), 1,4-dioxane-degrading *Mycobacterium dioxanotrophicus* (He et al., 2017), hydrogen-producing *Ethanoligenens harbinense* (Li et al., 2019), and ammonia-oxidizing archaeon *Candidatus Nitrosocosmicus franklandus* (Lehtovirta-Morley et al., 2016). In the transition network, six hub species included mushroom pathogenic *Janthinobacterium agaricidamnosum* (Graupner et al., 2015), human pathogenic *Raoultella ornithinolytica* (Morais et al., 2009), ochratoxin A-producing *Aspergillus homomorphus* (Varga et al., 2011), riboflavin-producing *Eremothecium gossypii* (Ledesma-Amaro et al., 2015), *Curtobacterium* sp. csp3, and *Micromonospora tulbaghiae*, whereas two bacterial species of oil hydrocarbon-degrading *Pedobacter cryoconitis* (Margesin et al., 2003) and *Flavobacterium branchiophilum* were the hubs in the conventional network.

### Beneficial Microbes Were Identified as Core Species and Agricultural Practices Induced Specific Microbial Compositions

Core microbes were identified by selecting the most abundant (detection) and ubiquitous (prevalence) taxa across all samples. We observed 28 bacteria and 32 fungi as core species of sugarcane phyllosphere, accounting for 0.9% of total observed taxa and 19.4% of overall relative abundance (Supplementary Figure 8). These core bacterial species consisted of plant growth-promoting bacteria [i.e., *Bacillus cereus* (Zhang et al., 2019), *B. thuringiensis* (Raddadi et al., 2008), *Pseudomonas aeruginosa* (Radhapriya et al., 2015), *Rhodopseudomonas palustris* (Xu et al., 2018), and *Methylobacterium radiotolerans* (Eevers et al., 2015)], antibiotic-producing *Streptomyces venezuelae* (Kim et al., 2019), cellulolytic *Sorangium cellulosum* (Wang et al., 2012), photosynthetic *Scytonema* sp. NIES-4073 (Will et al., 2019), catechol-degrading *P. resinovorans* (Nojiri et al., 2002), indole acetic acid (IAA)-producing *Acinetobacter baumannii* (Lin et al., 2018), and several animal- and human-associated pathogens. Fungal core taxa were dominated by four species of *Aspergillus* including *Aspergillus niger, A. neoniger, A. thermomutatus*, and *A. heteromorphus*, followed by two species of *Fusarium* such as *Fusarium proliferatum* and *F. oxysporum.* Similar to bacterial core species, several human pathogenic fungi as well as phytopathogens were also members of the core species in the sugarcane phyllosphere.

**FIGURE 6:**
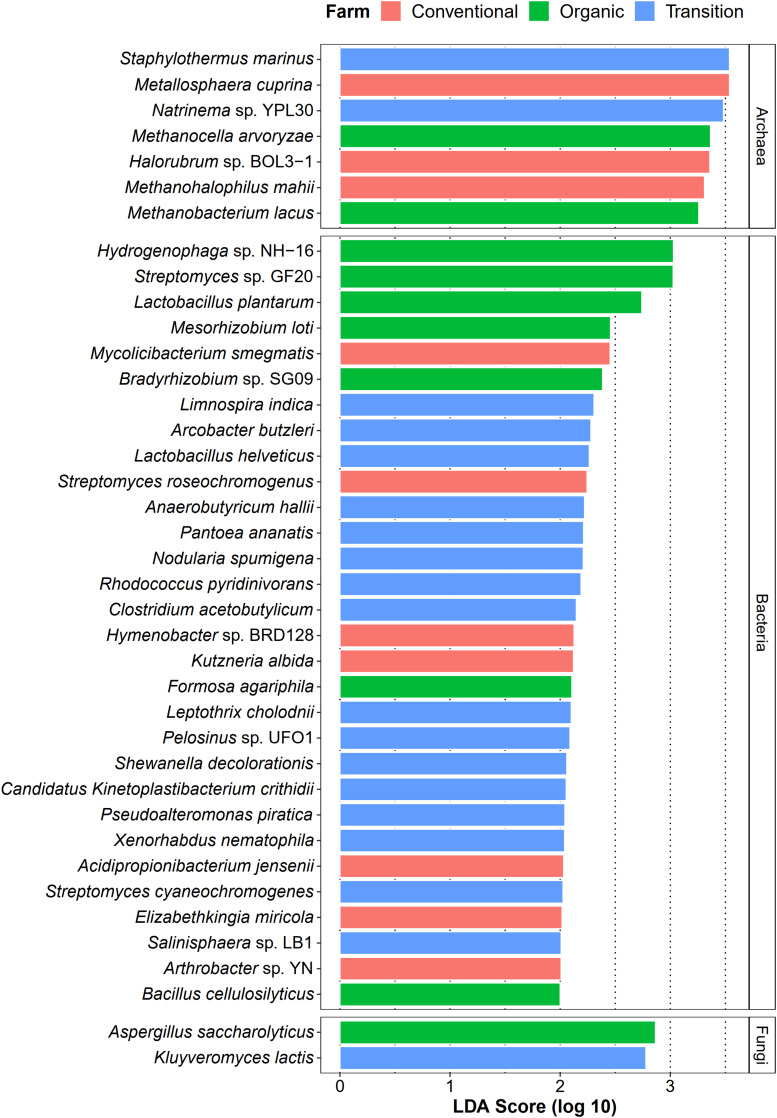
LEfSe analysis of taxonomic abundance among organic (green), transition (blue), and conventional (red) farming practices at the species level.

Apart from identifying common species of sugarcane phyllosphere, we also investigated whether farming practices could promote the abundance of specific taxa as their representatives (microbial signatures). In this case, LEfSe analysis (LDA score > 2.0) was used to statistically detect any microbial species of which their abundances were significantly different among farming practices (farming-specific taxa). In terms of the bacterial community, transition farming had the highest number of enriched taxa (16 species), while organic and conventional farms equally hosted 7 unique bacterial species (Figure 6). These bacterial species included plant growth-promoting bacteria, xenobiotic degraders, and pathogens. For the archaeal domain, seven species were identified, including three associated with conventional farming, two with organic farming, and two with transition farming (Figure 6). Furthermore, only two fungal taxa were observed, specifically one species in both organic and transition farms (Figure 6). However, no farming-specific fungi were observed in conventional farming.

### Highly Conserved and Unique Microbial Gene Functions Were Observed in Each Farming Practice

Similarly, we also noticed that the sugarcane phyllosphere microbial community shared common functions across all three farming practices. Specifically, 32 KOs were identified as core functions from overall samples (Supplementary Figure 9). Among them, genes associated with oxidative phosphorylation and photosynthesis were the most abundant, including F-type H^+^/Na^+^-transporting ATPase subunit alpha and beta (K02111 and K02112), F-type H^+^-transporting ATPase subunit alpha (K02132), cytochrome c oxidase subunit 1 (K02256), photosystem I P700 chlorophyll a apoprotein A1 and A2 (K02689 and K02690), photosystem II P680 reaction center D1 protein (K02703), nitrite reductase (NADH)-ubiquinone oxidoreductase chain 5 (K03883), NADH dehydrogenase (ubiquinone) Fe-S protein 2 (K03935), NAD(P)H-quinone oxidoreductase subunit 2 (K05573), and ribulose-bisphosphate carboxylase large chain (K01601). Genes involved in stress responses were also members of core functions such as heat shock 70 kDa protein 1/2/6/8 (K03283), molecular chaperone DnaJ (K03686), molecular chaperone HtpG (K04079), and a calcium permeable stress-gated cation channel (K21989).

**FIGURE 7:**
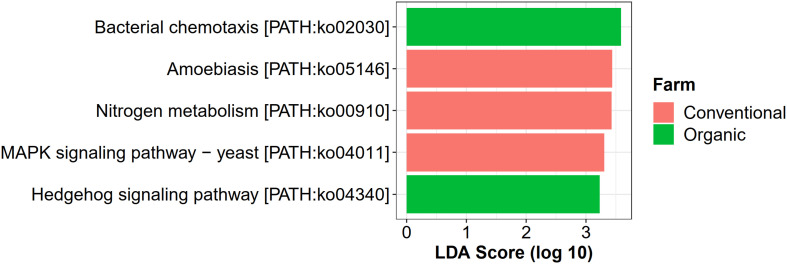
LEfSe analysis of functional abundance based on the KEGG pathway in all farming practices.

For farming-specific microbial functions, 31 unique KOs in total were identified by the LEfSe program with LDA score > 2.0, including 12 KOs associated with organic farming, 11 with conventional farming, and 8 with transition farming (Supplementary Figure 10). At the KEGG pathway level, bacterial chemotaxis (ko02030) and a hedgehog signaling pathway (ko04340) were uniquely enriched in organic farming, while three farming-specific pathways in conventional farming included amoebiasis (ko05146), nitrogen metabolism (ko00910), and MAPK signaling pathway-yeast (ko04011) (Figure 7). However, no significantly abundant pathway was found in transition farming.

## Discussion

Phyllosphere is described as the aerial parts of plant organs dominated by leaves (Turner et al., 2013). This habitat was tightly inhabited by up to 1 × 10^8^ bacterial cells g^–1^ fresh weight of leaves (Remus-Emsermann et al., 2014), and these associated microbiota were suggested to have great importance on the survivability of the host plants (Vorholt, 2012; Peñuelas and Terradas, 2014). In the present study, shotgun metagenome sequencing was used to investigate the effect of agricultural shift on taxonomic diversity and functional capacity of sugarcane phyllosphere microbial community. This approach directly sequences the entire genomic DNA in a sample, thus providing a minimally biased quantification of the absolute abundance of microbes.

Read-based taxonomic profiling exhibited that the composition of phyllosphere microbial community was mainly composed of the bacterial phyla Proteobacteria, Actinobacteria, and Firmicutes, and the fungal phyla Ascomycota and Basidiomycota in all three farming practices. The predominant bacterial and fungal phyla observed in this work were similar to the previously reported study of sugarcane using 16S rRNA and ITS amplicons (Hamonts et al., 2018), as well as in other plant species including tomato (Toju et al., 2019), and grapevine (Perazzolli et al., 2014). These results supported the earlier finding of highly conserved phyllosphere microbial communities across host species (Massoni et al., 2020).

Diversity analyses revealed that farming practices significantly influenced the taxonomic and functional profiles of phyllosphere microbiota (β-diversity). Organic farming was slightly more diverse (Shannon index) than transition and conventional farms, while this practice was also the lowest in terms of species evenness (Pielou index). Theoretically, more diverse microbial communities would potentially increase the resistance of the host plant to pathogen invasion with the support of beneficial microbial symbionts (Kinkel et al., 2011). *Streptomyces* sp. GF20 and *Lactobacillus plantarum* were identified as microbial signatures of organic farming by LEfSe, and these bacteria were also among the top 100 most abundant taxa in the overall organic samples. It indicated that these species might contribute to the decrease in the evenness index through their dominancy, which would be useful for the host performance. Notably, a similar response has been observed on the soil microbial community as reported by the previous research (Hartmann et al., 2014), indicating that above-ground microbes responded to agricultural managements in the same way as those colonizing below-ground compartments. The Shannon diversity index of functional potentials also tended to increase in organic farming compared with the other two farming practices. However, no statistical significance of taxonomic and functional α-diversities was detected. In addition, the district was also found to have a significant effect on both taxonomic and functional β-diversities. Although the influence of farming practice was stronger than that of the district based on the PERMANOVA *p*-value, the farming practice was not the sole factor structuring the composition and functional potentials of phyllosphere microbial communities.

Core microbiome was thought to be tightly associated and evolved with terrestrial plants for the long term (Yeoh et al., 2017), in which their gene functions were essential for plant growth and productivity (Lemanceau et al., 2017). Core species of sugarcane phyllosphere were comprised of plant growth-promoting bacteria including N_2_-fixing, antibiotic-producing, and IAA-producing species, highlighting the important roles of phyllosphere microbes to the fitness of host plants. Many of them have been cultivated *in vitro* (Raddadi et al., 2008; Radhapriya et al., 2015; Xu et al., 2018; Zhang et al., 2019). The presence of ammonia-oxidizing, cellulolytic, and photosynthetic bacteria as core members of sugarcane phyllosphere microbes further indicated their contributions to global nitrogen and carbon cycles, while the existence of a catechol-degrading bacterium showed phylloremediation potentials. For fungi, members of *Aspergillus* and *Fusarium* were the most predominant core taxa. Fungal species of *Aspergillus* have been studied for their plant growth-promoting properties including *Aspergillus niger*, which can solubilize phosphorous (P) (Mendes et al., 2015) and potassium (K) (Lopes-Assad et al., 2010). In contrast, *Fusarium* is one of the devastating phytopathogens that can adapt to a wide range of environments and affects various economic crops worldwide (Perincherry et al., 2019). Notably, by linking taxonomy and function, we found that beneficial genes possessed by core species mentioned above were commonly found in all samples. However, some of these genes were more abundant in organic and transition farms, while they were decreased in the conventional farm. Altogether, these core microbes emphasized the essential roles of phyllosphere microbial communities in ensuring plant growth and productivity, leading to the enhancement of the health of the entire host ecosystem.

Similar to the previous report of neotropical trees (Lajoie et al., 2020), conserved metabolic functions of phyllosphere microbes were also found in this work. Specifically, amino acid and carbohydrate metabolisms were enriched in all samples, indicating that these two functions were of prominent importance for the phyllosphere microbes to access the nutrients in this habitat (Müller et al., 2016), in that polysaccharides and proteins were parts of leaf exudates secreted by glandular trichomes, which also functioned in plant–microbe interactions (Schlechter et al., 2019). At the level of KO, oxidative phosphorylation, photosynthesis, and stress responses represented core phyllosphere microbial functions. It is thus not surprising that photosynthetic genes were part of the core functions as many core microbial species were photosynthetic bacteria. This also suggested that phyllosphere microbes might use light as an alternative source of energy upon nutrient deficit (Vorholt, 2012; Stiefel et al., 2013). Furthermore, a highly dynamic and extreme condition of the phyllosphere environment, including exposure to UV light and high temperatures, which varied during the entire day, enforced phyllosphere microbes to activate a number of genes associated with stress responses to successfully colonize and thrive on the leaf surface (Vorholt, 2012). In this study, we found numerous genes encoding heat-shock proteins as core functions, indicating that these genes might play critical roles in the adaptation and survival of phyllosphere microbes in this harsh habitat. These results were in accordance with previous studies that reported conserved functions related to phototrophy and microbial adaptation of phyllosphere microbial communities in different types of plants (Finkel et al., 2016; Lambais et al., 2017; Flores-Núñez et al., 2020).

Co-occurrence network analysis was subsequently performed to compare the structure and complexity of phyllosphere microbial communities in response to farming practices. Network topology assessments showed that organic network had the highest number of edges, density, and connectivity, which might be linked to the strong resilience capabilities of the phyllosphere microbiota under this farm to disturbances or invasion of exogenous microbes (Mendes et al., 2018; Banerjee et al., 2019; Liao et al., 2019). Moreover, greater complexity and connectivity in the organic network were followed by the number of hub species identified, while unsustainable farming was in contrast. This pattern was similar to the previous study of wheat root microbiome (Banerjee et al., 2019). Interestingly, the three farming practices harbored completely distinct hub species, illustrating that agricultural conversion shifted the central taxa in the community. Hub species of the organic network were dominated by *Streptomyces* spp., which are well-known antibiotic-producing bacteria (Kong et al., 2019). Most of the edges associated with Streptomyces hubs were positive (56 positive edges and 3 negative edges), including inter- and intraspecies associations. This indicated that *Streptomyces* could coexist and inhibit the growth of other microbes in regulating the composition and structure of the organic network. Hu et al. (2020) suggested that *Streptomyces* application could promote the growth of tomato by reshaping the rhizosphere microbial community and could positively increase the abundance of other beneficial bacteria. Cyanobacterial *Calothrix* sp. 336/3, which had abilities to fix atmospheric carbon and nitrogen (Kosourov et al., 2016), was the most connected node in the organic network, followed by polysaccharide-degrading *Lacinutrix* sp. 5H-3-7-4 (Klippel et al., 2011) and ammonia-oxidizing archaeon *Candidatus Nitrosocosmicus franklandus* (Lehtovirta-Morley et al., 2016). With their capabilities, these three hub species could be placed at a higher trophic level and were positively connected with the *Streptomyces* hubs, explaining the essential role of *Streptomyces*, which could therefore be assigned as potential keystone species in the organic network. In the transition network, bacterial pathogens of mushroom, plant, and human were the hub species, while the conventional network had the lowest number of hub taxa, comprising of oil hydrocarbon-degrading *Pedobacter cryoconitis* (Margesin et al., 2003) and pathogenic *Flavobacterium branchiophilum* (Good et al., 2015). In short, the identified keystone species of phyllosphere microbes might play important ecological roles in their respective agricultural practices, which provided a future direction of study. However, further experimental validation regarding the biological process governing microbial networks is required, such as using a synthetic community.

Differential abundant analysis revealed that conventional practice induced the abundance of pathogenic species and microbes that could tolerate the harsh environments, while organic farming was characterized by the enrichment of beneficial species for the host plant. Conventional-specific taxa comprised of metal-mobilizing *Metallosphaera cuprina* (Liu et al., 2011), environmental extremes-resistant *Halorubrum* sp. BOL3-1 (DasSarma et al., 2019), and human pathogenic bacteria such as *Elizabethkingia miricola* (Zdziarski et al., 2017), and *Mycolicibacterium smegmatis* (Yamada et al., 2018). Organic-specific taxa consisted of plant growth-promoting bacteria including N_2_-fixing bacteria [i.e., *Mesorhizobium loti* (Shimoda et al., 2016) and *Bradyrhizobium* sp. SG09 (Wasai-Hara et al., 2020)], lactic acid bacterium *L. plantarum*, which had the capability to behave as a biocontrol agent against phytopathogens (Daranas et al., 2019), and cellulase-degrading *Bacillus cellulosilyticus* (Mead et al., 2013). The number of transition-specific taxa was the highest, suggesting that many microbes might act as intermediate agents of agricultural transformation from conventional practices to organic systems. These species were dominated by toxic compound degraders including pyridine-degrading *Rhodococcus pyridinivorans* (Yoon et al., 2000), dye-decolorizing *Shewanella decolorationis* (Xu et al., 2005), metal-reducing *Pelosinus* sp. UFO1 (Brown et al., 2014), Fe(II)-oxidizing *Leptothrix cholodnii* (Kunoh et al., 2016), as well as phytopathogenic *Pantoea ananatis* (Stice et al., 2018), and enteropathogenic *Arcobacter butzleri* (Prouzet-Mauléon et al., 2006). One possible explanation was that residues of applied agrochemicals in the previous practice of transition farming remained in the soil and were absorbed by plants and then distributed to the leaves (Myers et al., 2016). As a response, the abundance of these microbes was increased, which could help to remediate those toxic substances. Thus, these beneficial sugarcane microbiomes could be utilized as a promising approach for accelerating the conversion of conventional farming to organic management. Remarkably, several species belonging to genus *Streptomyces* were found in all farming practices by LEfSe analysis, identified as core species across all samples, and assigned as potential keystone species in the organic network. It thus suggested that *Streptomyces* spp. were well-adapted taxa in various conditions and might play crucial roles in the sugarcane growth and productivity (Sousa and Olivares, 2016; Wang et al., 2019).

Besides driving microbial composition, farming practices also significantly affected cell motility and energy metabolism of phyllosphere microbes. Cell motility was predominant in organic farming, particularly bacterial chemotaxis, suggesting that the active movement of microbial species in organic farming was greater than those in transition and conventional farms. Chemotaxis signaling allowed motile phyllosphere microorganisms to exploit chemical gradients to find nutrient resources such as leaf exudates released by the host plant or to escape noxious compounds (Scharf et al., 2016). Thus, the limitation of nutrients on the leaf surface made this property critical for the epiphytes to survive in this oligotrophic habitat (Vorholt, 2012; Scharf et al., 2016). Energy metabolism by means of the photosynthetic pathway was the highest in transition farming followed by organic farming and was significantly decreased in conventional farming, suggesting that higher energy was required to transform the agrochemical-polluted land into healthier farmland. This also indicated that the application of agrochemicals, especially herbicides, in the conventional practice reduced the photosynthetic potentials of the overall phyllosphere microbial communities (Broser et al., 2011; Crouzet et al., 2019). Furthermore, fertilization in the conventional farm could also alter the composition of leaf exudates (Barunawati et al., 2013), which might not favor its associated phyllosphere colonizers. In addition, valine, leucine, and isoleucine biosynthesis, also known as branched-chain amino acids (BCAAs), was also depleted in conventional farming. BCAAs were global transcriptional regulators in bacteria, which were importantly involved in the response to nutrient availability and metabolic reprogramming upon nutrient depletion (Kaiser and Heinrichs, 2018). Taken together, these might be the reason for the decrease in microbial diversity in conventional farming as some of the phyllosphere microbes lose these essential abilities to grow and survive.

Conventional farming, in contrast, slightly increased the abundance of xenobiotics biodegradation and metabolism, while organic farming was the lowest. This supported the idea that conventional farming, which relied on synthetic agrochemical usages (i.e., herbicides and NPK fertilizers), provided a toxic environment for the microbes, and xenobiotic-degrading genes were increased as a response (Liao et al., 2019). It also elevated the nitrogen metabolism of the phyllosphere microbes, including glutamine synthetase (K01915) and nitrite reductase (NADH) large subunit (K00362), which were identified as conventional-specific functions by LEfSe. The latter was reported to participate indirectly in nitric oxide (NO) production through the denitrification process (Härtig and Zumft, 1999; Ruiz et al., 2019). Interestingly, human pathogenic genes involved in the amoebiasis pathway were specifically enriched under conventional farming, emphasizing the harmful effect of unsustainable practices not only to the environment but also to human health.

While it was apparent that farming practices significantly influenced the taxonomic diversity and functional capacity of phyllosphere microbial communities, our experimental design in which triplicate samples of a particular farming type were collected from the same field could not clearly separate the effects of agricultural practices and farmland areas. Therefore, the study of the impacts of geographical location with similar farming practices, including soil properties and environmental and seasonal conditions, is warranted in the future to elucidate the essential roles of phyllosphere microbial communities for agricultural conversion toward sustainable farming.

## Conclusion

This study revealed that organic farming promoted a higher microbial diversity with a more complex and stable network structure. Greater numbers of plant growth-promoting bacteria were also enriched as farming-specific taxa in this practice. Transition farming was predominated by numerous toxic compound degraders, which might importantly contribute to the restoration of damaged agroecosystems such as remediation of agrochemical residues. Conventional farming, which had the lowest diversity index, increased the abundance of extremes-resistant and human pathogenic species. Decreased microbial diversity in conventional farming might be attributed to diminished cell motility and energy metabolism, which were essentially required for the phyllosphere microbes to survive and thrive under stressful conditions in the phyllosphere habitat, including nutrient limitation. Overall, our results highlighted that shifting agricultural managements induced community changes and distinct functional profiles of phyllosphere microbial community, demonstrating the potential use of phyllosphere microbiota as signatures for improving agrochemical-derived farming to healthier and more sustainable systems. In addition, our work provided a basis for future research on the response of sugarcane-associated phyllosphere microbiota to agricultural land conversion and their prospects for enhancing crop productivity and global ecosystem health.

## Data Availability Statement

The datasets presented in this study can be found in online repositories. The names of the repository/repositories and accession number(s) can be found below: https://www.ebi.ac.uk/ena, PRJEB38470.

## Author Contributions

AK, SW, and SC designed the experiment. AK, JJ, AT, PP, and SD performed sample collection. AK and SD conducted DNA extraction. SW, JJ, and SD arranged the sequencing process. AK, SS, and KK carried out the shotgun metagenome data analysis. AK, SC, SS, and SW contributed to data interpretation. AK wrote the first draft of the manuscript. All authors critically revised and approved the final version of the manuscript to be published.

## Conflict of Interest

The authors declare that the research was conducted in the absence of any commercial or financial relationships that could be construed as a potential conflict of interest.

## Supplementary Material

Supplementary Figure 1 Map of sampling locations created with Google maps.

Supplementary Figure 2 Relative abundance of the KEGG major category visualized by histogram in three different farming practices.

Supplementary Figure 3 Relative abundance of the KEGG category from the highest (top) to the lowest (bottom) in three different farming practices.

Supplementary Figure 4 Top 25 relative abundances of the KEGG pathway in three different farming practices.

Supplementary Figure 5 α-Rarefaction curves, drawn based on the abundance of microbial communities in conventional, organic, and transition farming practices represented by red, green, and blue, respectively.

Supplementary Figure 6 Top 50 most influential microbial species to the community differences among farming practices. The *x*-axis values are based on the coefficient score of the PERMANOVA test. The *y*-axis (microbial species) highlighted by green color are microbial signatures of organic farming identified by LEfSe.

Supplementary Figure 7 Keystone species observed in the three farming-specific microbial co-occurrence networks. The table shows the node degree of each taxon. Rows represent microbial species and columns represent farming practices. Horizontal and vertical dendrograms were constructed by the hierarchical clustering method.

Supplementary Figure 8 Core phyllosphere microbes of sugarcane sampled from three different farming practices.

Supplementary Figure 9 Core gene functions of phyllosphere microbes associated with sugarcane in organic, transition, and conventional farming practices.

Supplementary Figure 10 LEfSe test of functional abundance based on KEGG orthology (KO) in organic, transition, and conventional farming practices.

Supplementary Table 1 Description of three sugarcane farming practices sampled in this study.

Supplementary Table 2 Number of reads remaining before and after QC processes.

Supplementary Table 3 Good’s coverage values of all samples in three farming practices.

Supplementary Table 4 PERMANOVA and BETADISPER analysis results.

Supplementary Table 5 Co-occurrence network attributes in three different farming practices.
