# Supplementary material for: Comparative Metagenomics Reveals Microbial Signatures of Sugarcane Phyllosphere in Organic Management
Source: Front Microbiol. 2021 Mar 22;12:623799. doi: 10.3389/fmicb.2021.623799 (PMC8019924; doi:10.3389/fmicb.2021.623799)

Sampling map

Farm

- Organic farm (OP)
- Transition farm (TP)
- Conventional farm (CP)

Distance

± 4 km between OP and TP
± 37 km between OP and CP
± 39 km between TP and CP

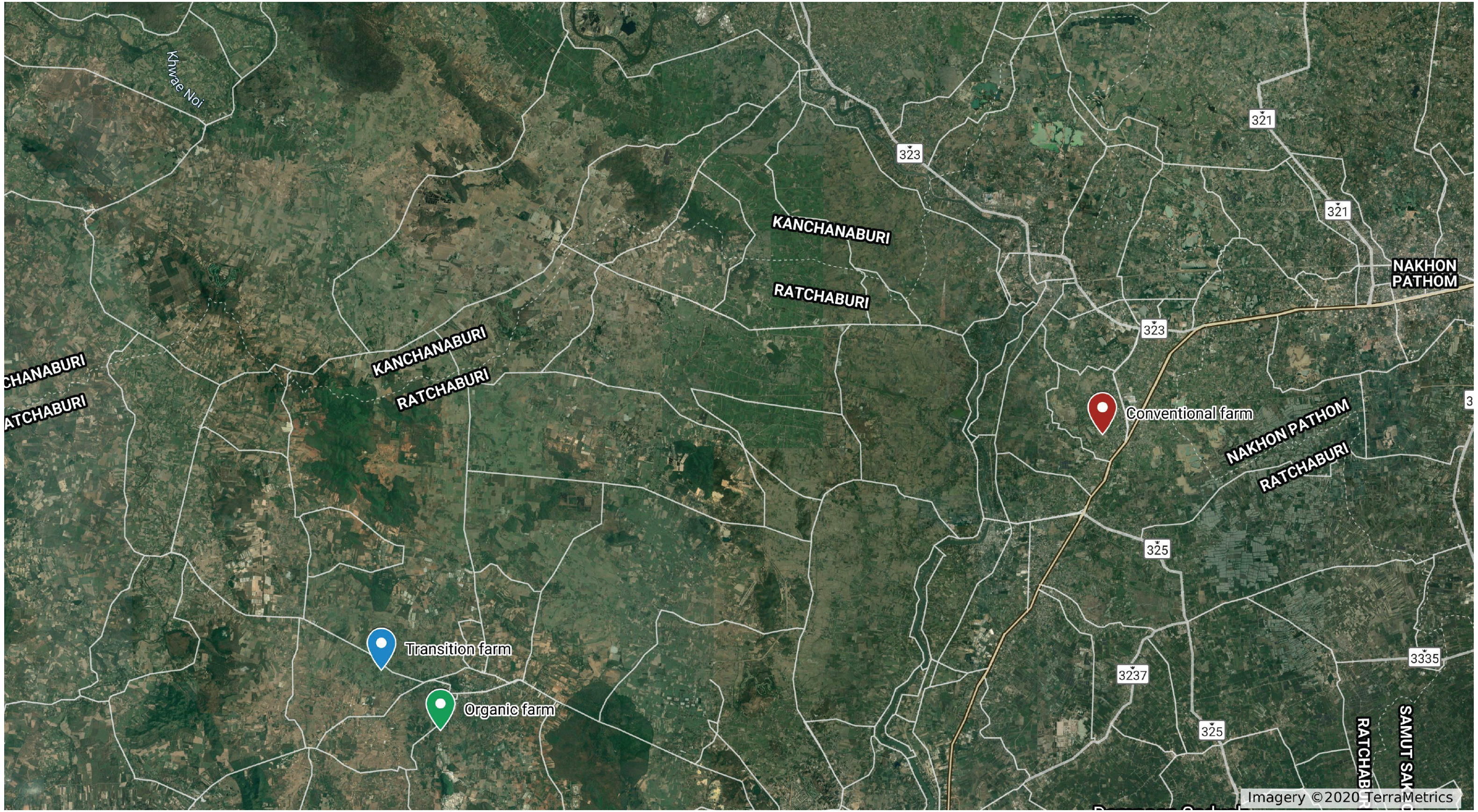

Supplement: Supplementary Figure 1 — Map of sampling locations created with Google maps. [file Image_1.PDF]
