# Supplementary figures and images for: Comparative Metagenomics Reveals Microbial Signatures of Sugarcane Phyllosphere in Organic Management
Source: Front Microbiol. 2021 Mar 22;12:623799. doi: 10.3389/fmicb.2021.623799 (PMC8019924; doi:10.3389/fmicb.2021.623799)

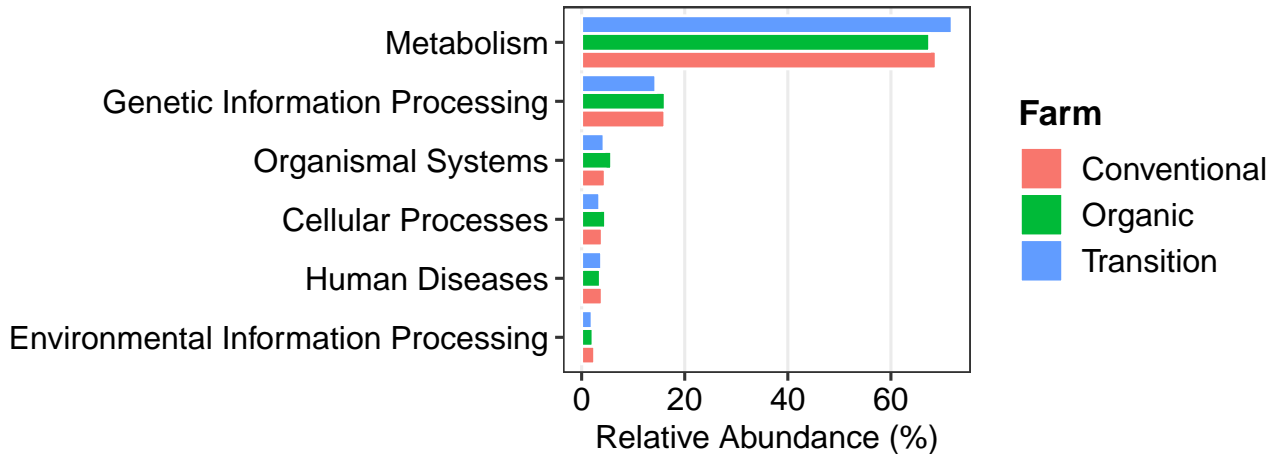

Supplement: Supplementary Figure 2 — Relative abundance of the KEGG major category visualized by histogram in three different farming practices. [file Image_2.PDF]

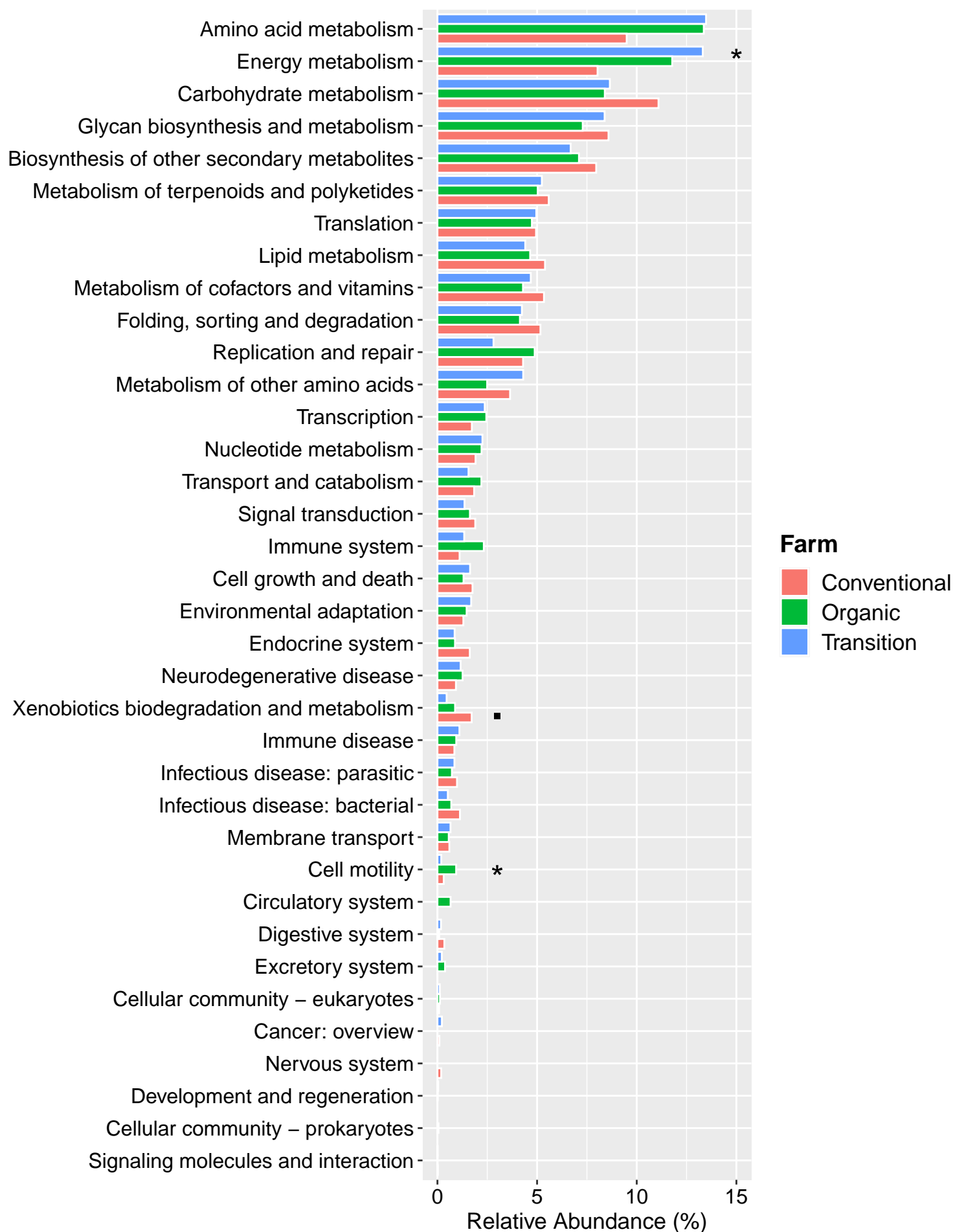

Supplement: Supplementary Figure 3 — Relative abundance of the KEGG category from the highest (top) to the lowest (bottom) in three different farming practices. [file Image_3.PDF]

Farm ■ Conventional ■ Organic ■ Transition

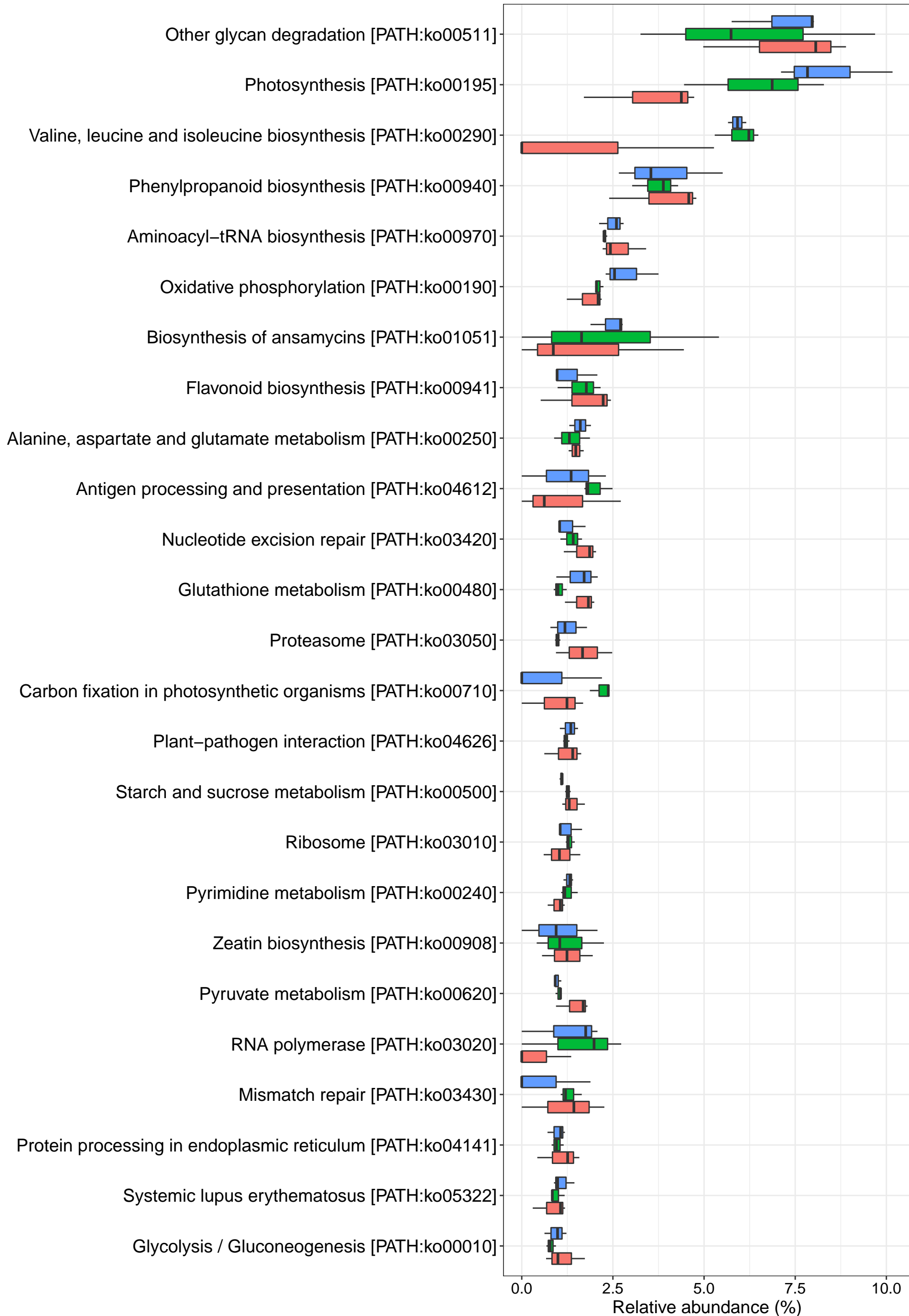

Supplement: Supplementary Figure 4 — Top 25 relative abundances of the KEGG pathway in three different farming practices. [file Image_4.PDF]

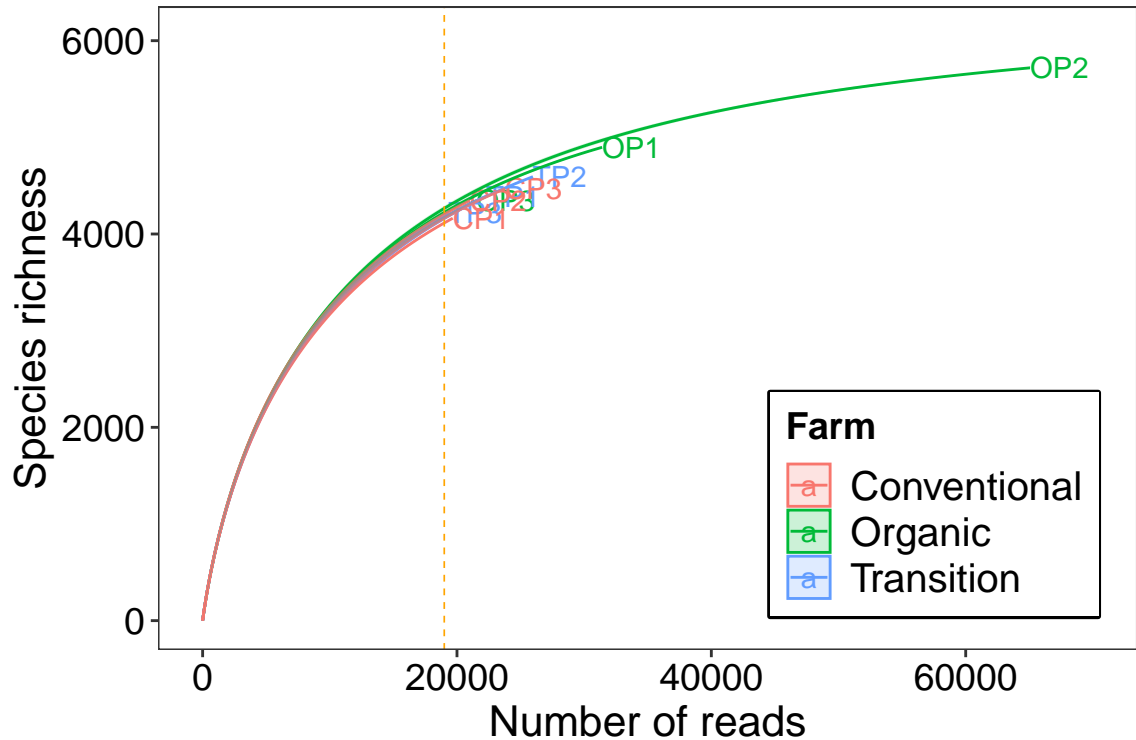

Supplement: Supplementary Figure 5 — α-Rarefaction curves, drawn based on the abundance of microbial communities in conventional, organic, and transition farming practices represented by red, green, and blue, respectively. [file Image_5.PDF]

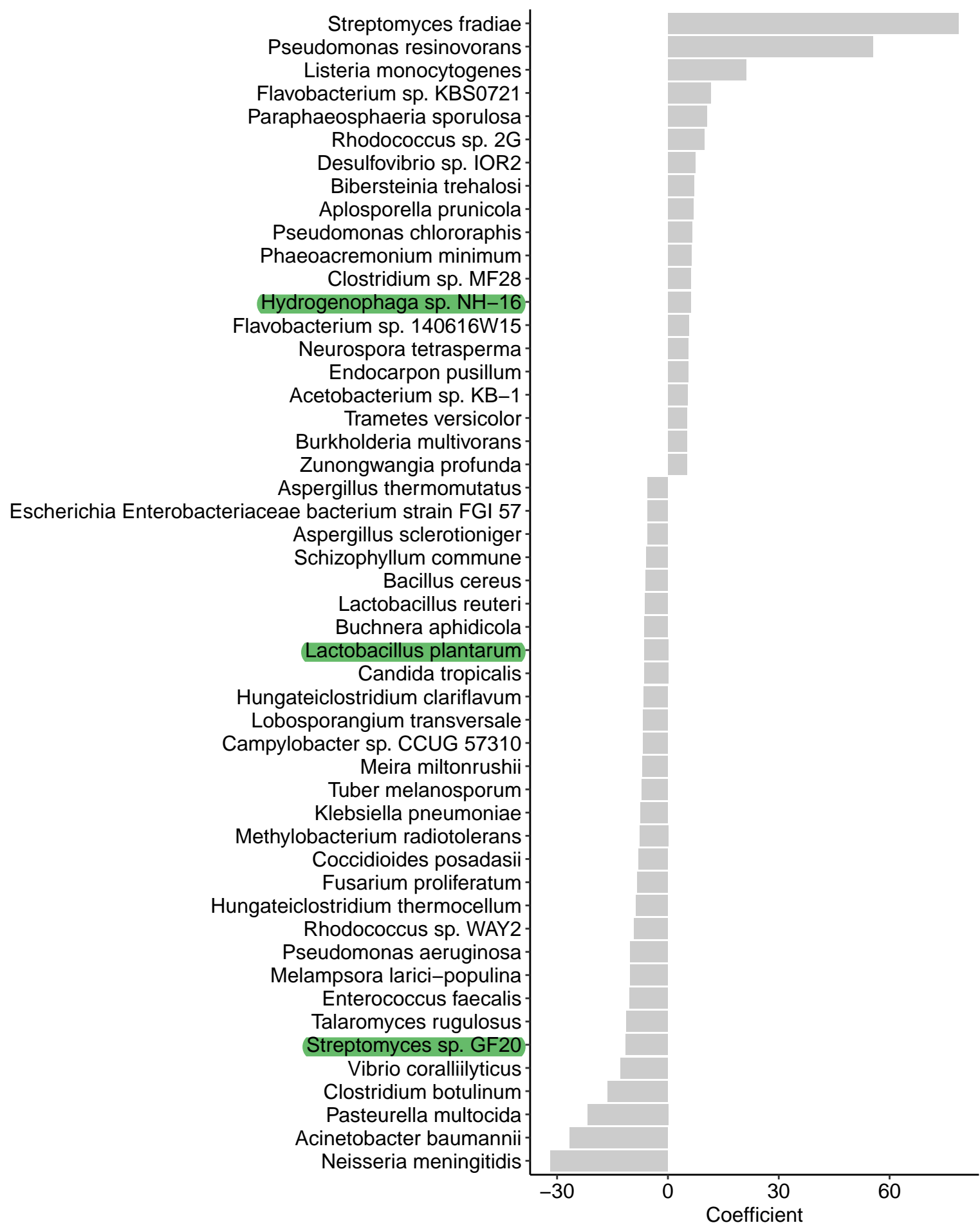

Supplement: Supplementary Figure 6 — Top 50 most influential microbial species to the community differences among farming practices. The x-axis values are based on the coefficient score of the PERMANOVA test. The y-axis (microbial species) highlighted by green color are microbial signatures of organic farming identified by LEfSe. [file Image_6.PDF]

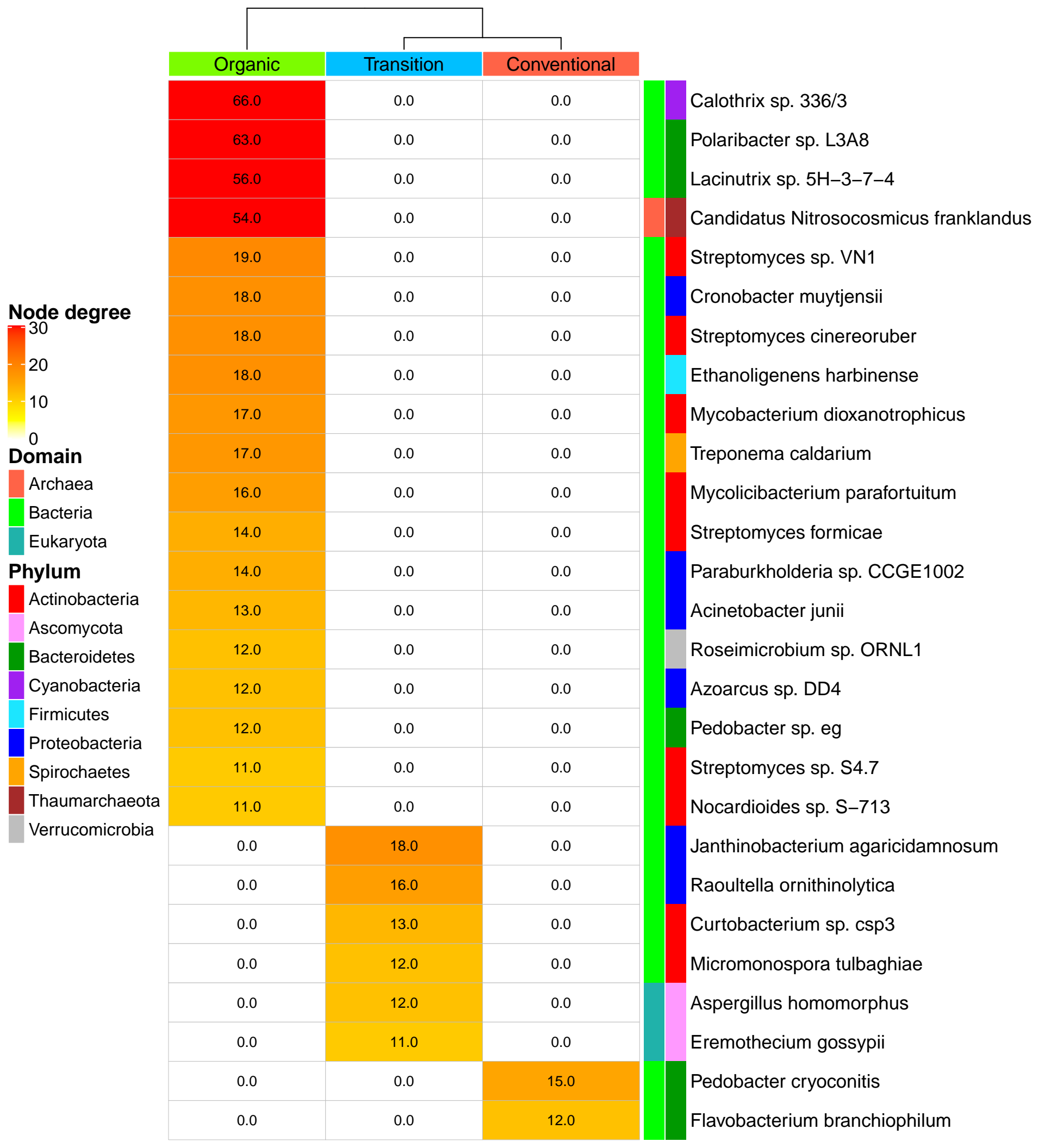

Supplement: Supplementary Figure 7 — Keystone species observed in the three farming-specific microbial co-occurrence networks. The table shows the node degree of each taxon. Rows represent microbial species and columns represent farming practices. Horizontal and vertical dendrograms were constructed by the hierarchical clustering method. [file Image_7.PDF]

Core

Taxa

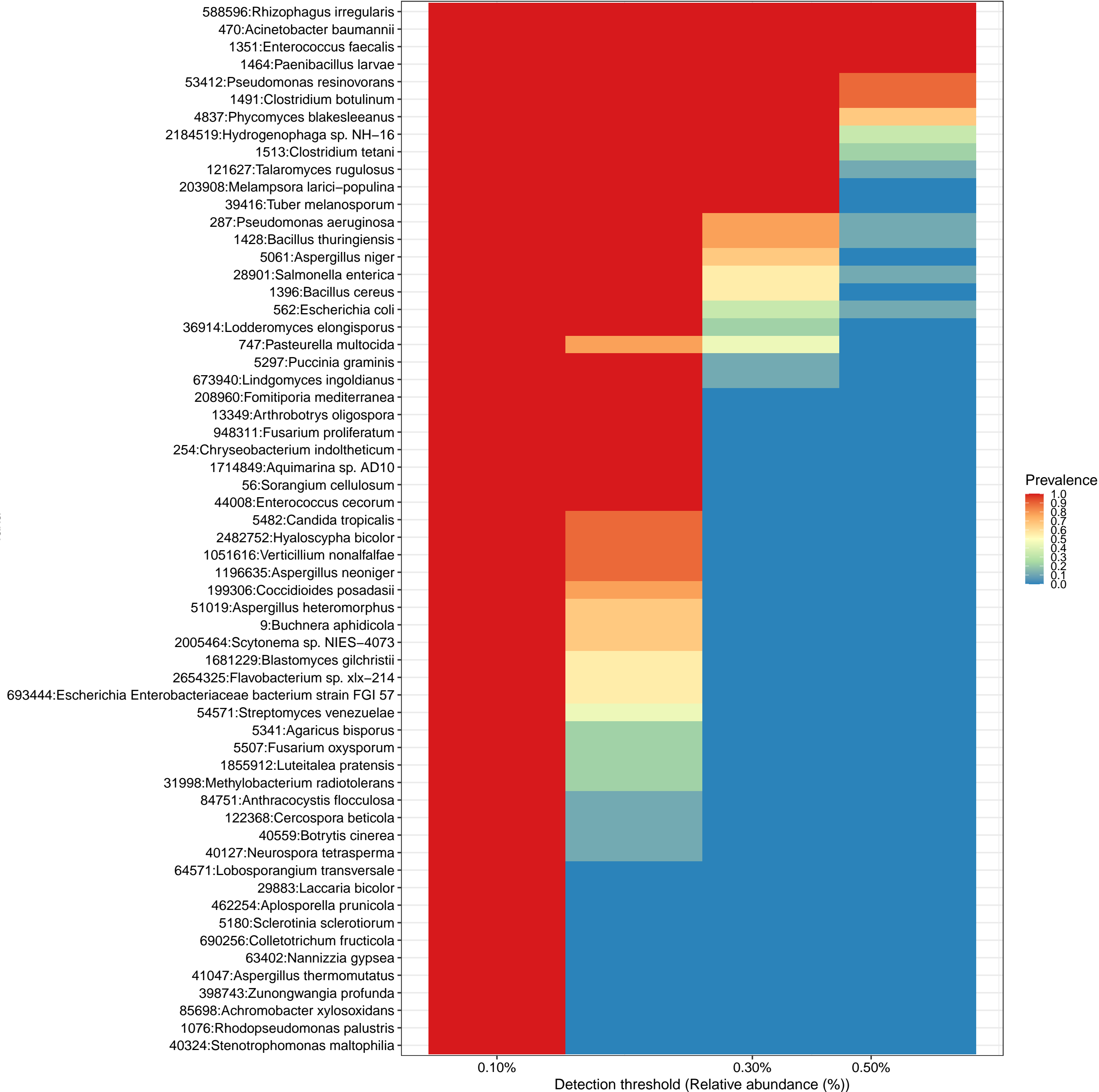

Supplement: Supplementary Figure 8 — Core phyllosphere microbes of sugarcane sampled from three different farming practices. [file Image_8.PDF]

Core function

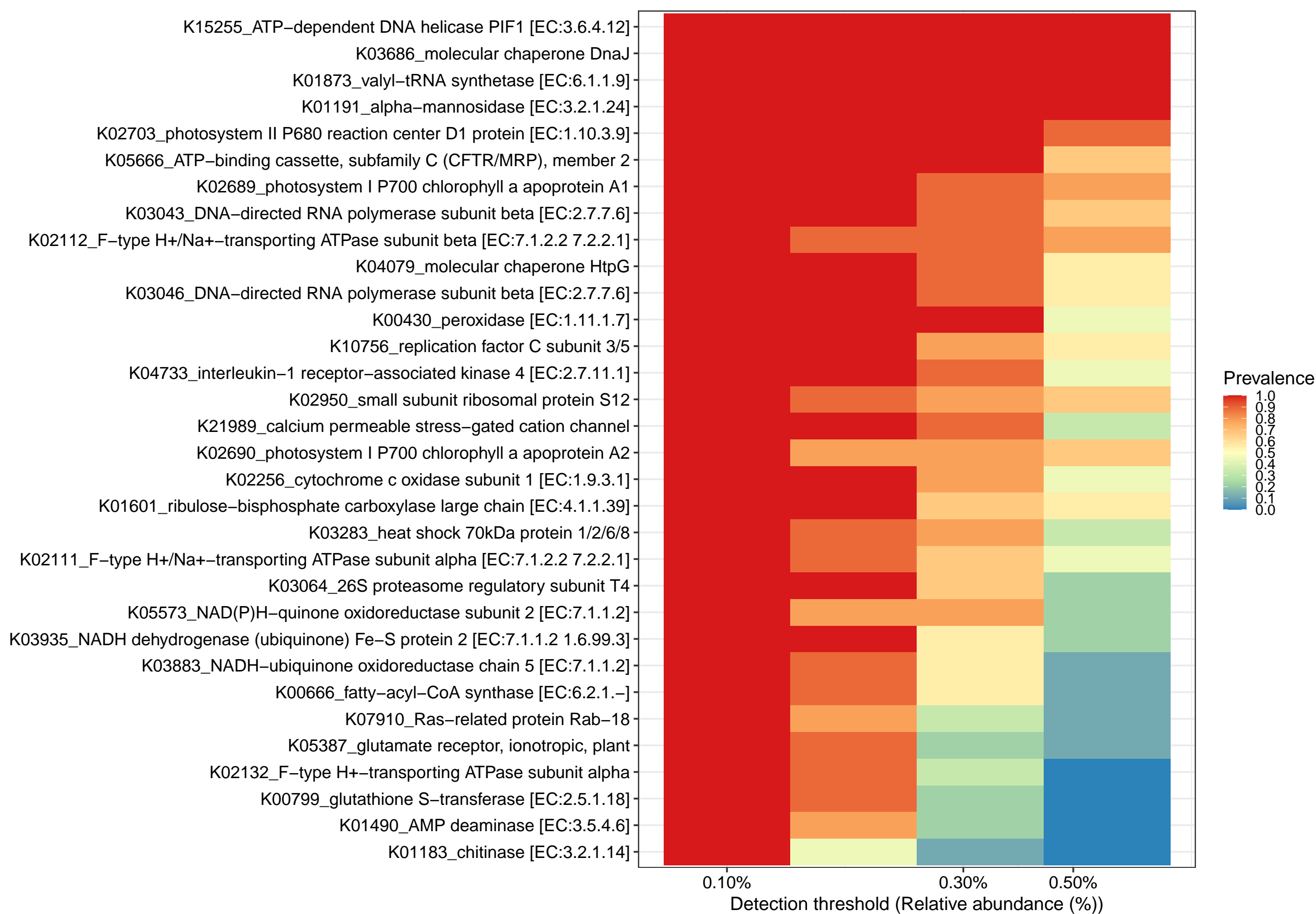

Supplement: Supplementary Figure 9 — Core gene functions of phyllosphere microbes associated with sugarcane in organic, transition, and conventional farming practices. [file Image_9.PDF]
