# Supplementary material for: Comparative Metagenomics Reveals Microbial Signatures of Sugarcane Phyllosphere in Organic Management
Source: Front Microbiol. 2021 Mar 22;12:623799. doi: 10.3389/fmicb.2021.623799 (PMC8019924; doi:10.3389/fmicb.2021.623799)
Supplement: Supplementary Figure 10 — LEfSe test of functional abundance based on KEGG orthology (KO) in organic, transition, and conventional farming practices. [file Image_10.PDF]

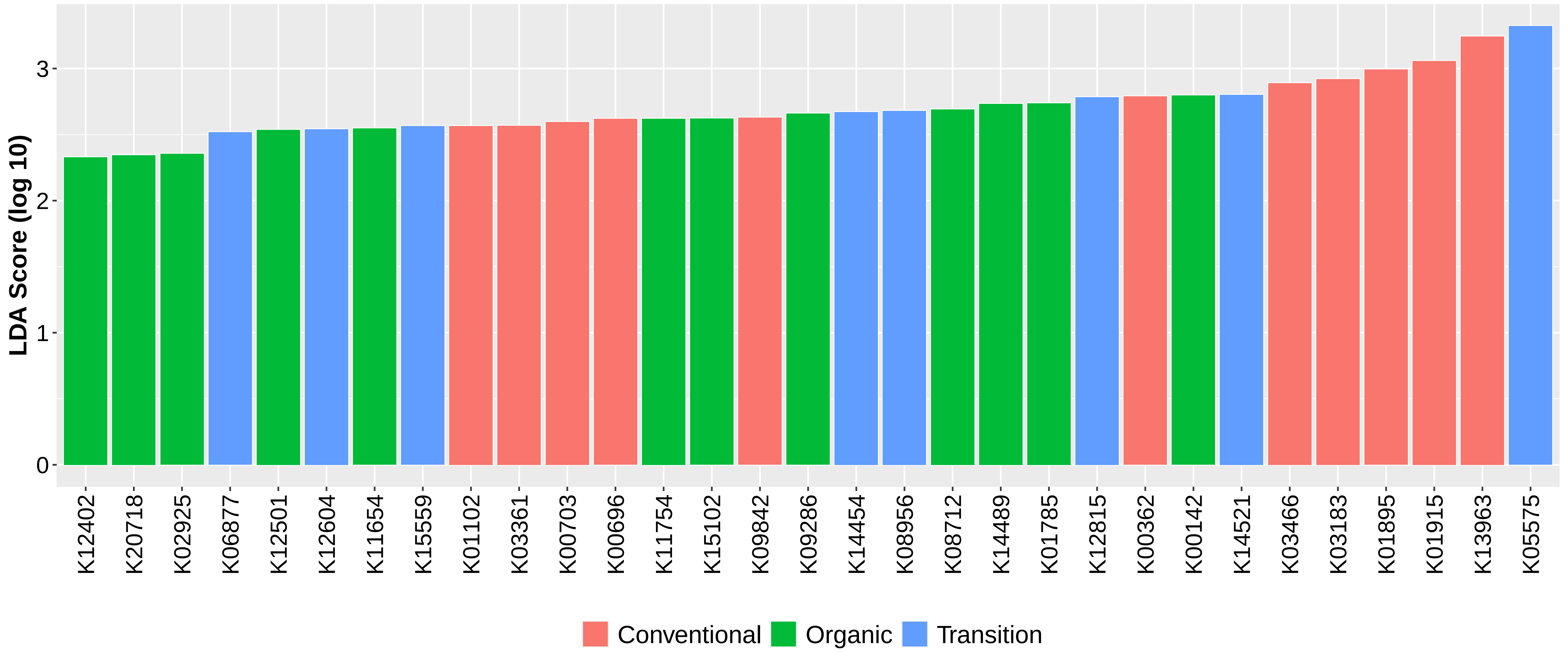

- K00142_acyl−CoA synthetase [EC:6.2.1.−]
- K00362_nitrite reductase (NADH) large subunit [EC:1.7.1.15]
- K00696_sucrose−phosphate synthase [EC:2.4.1.14]
- K00703_starch synthase [EC:2.4.1.21]
- K01102_pyruvate dehydrogenase phosphatase [EC:3.1.3.43]
- K01785_aldose 1−epimerase [EC:5.1.3.3]
- K01895_acetyl−CoA synthetase [EC:6.2.1.1]
- K01915_glutamine synthetase [EC:6.3.1.2]
- K02925_large subunit ribosomal protein L3e
- K03183_demethylmenaquinone methyltransferase / 2−methoxy−6−polyprenyl−1,4−benzoquinol methylase [EC:2.1.1.163 2.1.1.201]
- K03361_F−box and WD−40 domain protein CDC4
- K03466_DNA segregation ATPase FtsK/SpoIIIE, S−DNA−T family
- K05575_NAD(P)H−quinone oxidoreductase subunit 4 [EC:7.1.1.2]
- K06877_DEAD/DEAH box helicase domain−containing protein
- K08712_ATP−binding cassette, subfamily G (WHITE), member 2, SNQ2
- K08956_AFG3 family protein [EC:3.4.24.−]
- K09286_EREBP−like factor
- K09842_abscisic−aldehyde oxidase [EC:1.2.3.14]
- K11654_SWI/SNF−related matrix−associated actin−dependent regulator of chromatin subfamily A member 5 [EC:3.6.4.−]
- K11754_dihydrofolate synthase / folylpolyglutamate synthase [EC:6.3.2.12 6.3.2.17]
- K12402_AP−4 complex subunit mu−1
- K12501_homogentisate solanesyltransferase [EC:2.5.1.117]
- K12604_CCR4−NOT transcription complex subunit 1
- K12815_pre−mRNA−splicing factor ATP−dependent RNA helicase DHX38/PRP16 [EC:3.6.4.13]
- K13963_serpin B
- K14454_aspartate aminotransferase, cytoplasmic [EC:2.6.1.1]
- K14489_arabidopsis histidine kinase 2/3/4 (cytokinin receptor) [EC:2.7.13.3]
- K14521_N−acetyltransferase 10 [EC:2.3.1.−]
- K15102_solute carrier family 25 (mitochondrial phosphate transporter), member 3
- K15559_regulator of Ty1 transposition protein 103
- K20718_LRR receptor−like serine/threonine−protein kinase ERECTA [EC:2.7.11.1]
