# Supplementary material for: Comparative Metagenomics Reveals Microbial Signatures of Sugarcane Phyllosphere in Organic Management
Source: Front Microbiol. 2021 Mar 22;12:623799. doi: 10.3389/fmicb.2021.623799 (PMC8019924; doi:10.3389/fmicb.2021.623799)
Supplement: Supplementary Table 1 — Description of three sugarcane farming practices sampled in this study. [file Table_1.pdf]

Table S1. Description of three sugarcane farming practices sampled in this study

	Organic	Transition	Conventional
Location	Rai Sukphoang, Chom Bueng District, Ratchaburi	Rai Sarot, Rang Bua, Chom Bueng District, Ratchaburi	Rai Pramote, Photharam District, Ratchaburi
Coordinate	13°35'42.5"N 99°34'58.0"E	13°37'52.8"N 99°33'10.7"E	13°44'34.2"N 99°54'16.6"E
Plant age	10 months	10 months	10 months
Plant variety	Khon Kaen 3 (KK3)	Khon Kaen 3 (KK3)	Khon Kaen 3 (KK3)
Synthetic fertilizer	–	–	NPK fertilizer (MORAKOT, RISINGSUN)
Synthetic herbicide	–	–	2,4-D sodium salt 95% SP (P. Chemitech co., Ltd.)
Organic fertilizer	1. Swine manure<br>2. Fermented bamboo as bio-compost	Chicken manure	–

Note: “ – “ : Not applied
