# Supplementary material for: Comparative Metagenomics Reveals Microbial Signatures of Sugarcane Phyllosphere in Organic Management
Source: Front Microbiol. 2021 Mar 22;12:623799. doi: 10.3389/fmicb.2021.623799 (PMC8019924; doi:10.3389/fmicb.2021.623799)
Supplement: Supplementary Table 2 — Number of reads remaining before and after QC processes. [file Table_2.pdf]

Table S2. Number of reads remaining before and after QC processes

Sample	Raw reads	Host DNA (%)	Trimming and Filtering (%)	Microbial DNA (%)	Final reads
OP1	14,010,320	12,549,451 (89.6)	456,592 (3.3)	7.17	1,004,277
OP2	29,216,274	26,213,525 (89.7)	921,331 (3.2)	7.12	2,081,418
OP3	10,193,636	9,201,818 (90.3)	279,024 (2.7)	6.99	712,794
TP1	9,892,628	8,857,044 (89.5)	296,860 (3.0)	7.47	738,724
TP2	10,874,664	9,671,801 (88.9)	340,623 (3.1)	7.93	862,240
TP3	10,040,759	9,087,862 (90.5)	294,101 (2.9)	6.56	658,796
CP1	10,150,908	9,187,407 (90.5)	303,564 (2.9)	6.5	659,937
CP2	11,988,697	10,909,786 (91.0)	365,186 (3.1)	5.95	713,725
CP3	12,167,187	11,021,006 (90.6)	401,274 (3.3)	6.12	744,907
**Total**	118,535,073				8,176,818

Abbreviations: OP: Organic, TP: Transition, CP: Conventional, QC: Quality control
