# Supplementary material for: Comparative Metagenomics Reveals Microbial Signatures of Sugarcane Phyllosphere in Organic Management
Source: Front Microbiol. 2021 Mar 22;12:623799. doi: 10.3389/fmicb.2021.623799 (PMC8019924; doi:10.3389/fmicb.2021.623799)
Supplement: Supplementary Table 3 — Good’s coverage values of all samples in three farming practices. [file Table_3.pdf]

Table S3. Good’s coverage values of all samples in three farming practices

**Sample**	**Farming**	**No. singletons**	**No. sequences**	**Good’ coverage**
OP1	Organic	1,145	19,000	93.97368
OP2	Organic	1,307	19,000	93.12105
OP3	Organic	926	19,000	95.12632
TP1	Transition	971	19,000	94.88947
TP2	Transition	1,052	19,000	94.46316
TP3	Transition	921	19,000	95.15263
CP1	Conventional	902	19,000	95.25263
CP2	Conventional	930	19,000	95.10526
CP3	Conventional	960	19,000	94.94737
