# Supplementary material for: Comparative Metagenomics Reveals Microbial Signatures of Sugarcane Phyllosphere in Organic Management
Source: Front Microbiol. 2021 Mar 22;12:623799. doi: 10.3389/fmicb.2021.623799 (PMC8019924; doi:10.3389/fmicb.2021.623799)
Supplement: Supplementary Table 4 — PERMANOVA and BETADISPER analysis results. [file Table_4.pdf]

Table S4. PERMANOVA and BETADISPER analysis results

A. Taxonomic β-diversity

- PERMANOVA test with farming practices as the factor

	Df	SumsOfSqs	MeanSqs	F. Model	R^2	Pr(>F)
Farm	2	0.1321	0.066049	1.1125	0.27052	0.003 **
Residuals	6	0.35621	0.059368		0.72948	
Total	8	0.48831			1	

- PERMANOVA test with district (location) as the factor

	Df	SumsOfSqs	MeanSqs	F. Model	R^2	Pr(>F)
District	1	0.06934	0.06934	1.1585	0.142	0.011 *
Residuals	7	0.41897	0.059853		0.858	
Total	8	0.48831			1	

- BETADISPER test with farming practices as the factor

	Df	Sum Sq	Mean Sq	F	N. Perm	Pr(>F)
Group	2	0.00081	0.00041	7.2003	999	0.001 ***
Residuals	6	0.00033	0.00005			

- BETADISPER test with district (location) as the factor

	Df	Sum Sq	Mean Sq	F	N. Perm	Pr(>F)
Group	1	0.00017	0.00017	1.3106	999	0.285
Residuals	7	0.00095	0.00014			

B. Functional β-diversity

- PERMANOVA test with farming practices as the factor

	Df	SumsOfSqs	MeanSqs	F. Model	R^2	Pr(>F)
Farm	2	0.43295	0.21648	1.1859	0.28331	0.01 *
Residuals	6	1.09523	0.18254		0.71669	
Total	8	1.52818			1	

- PERMANOVA test with district (location) as the factor

	Df	SumsOfSqs	MeanSqs	F. Model	R^2	Pr(>F)
District	1	0.2696	0.2696	1.4995	0.17642	0.02 *
Residuals	7	1.2586	0.1798		0.82358	
Total	8	1.5282			1	

- BETADISPER test with farming practices as the factor

	Df	Sum Sq	Mean Sq	F	N. Perm	Pr(>F)
Group	2	0.0118	0.0059	2.586	999	0.186
Residuals	6	0.0137	0.0023			

- BETADISPER test with district (location) as the factor

	Df	Sum Sq	Mean Sq	F	N. Perm	Pr(>F)
Group	1	0.00242	0.00242	1.3554	999	0.268
Residuals	7	0.01249	0.00178			
