# Supplementary material for: Comparative Metagenomics Reveals Microbial Signatures of Sugarcane Phyllosphere in Organic Management
Source: Front Microbiol. 2021 Mar 22;12:623799. doi: 10.3389/fmicb.2021.623799 (PMC8019924; doi:10.3389/fmicb.2021.623799)
Supplement: Supplementary Table 5 — Co-occurrence network attributes in three different farming practices. [file Table_5.pdf]

Table S5. Co-occurrence network attributes in three different farming practices

	Organic	Transition	Conventional
Number of nodes	500	500	500
Number of edges	438	291	200
Modularity	0.289	0.649	0.633
Clustering coefficient	0.246	0.17	0.168
Density	0.003	0.002	0.001
Diameter	8	11	12
